# A Novel Polyphenol Oxidoreductase OhLac from *Ochrobactrum* sp. J10 for Lignin Degradation

**DOI:** 10.3389/fmicb.2021.694166

**Published:** 2021-10-04

**Authors:** Chenxian Yang, Lingling Ma, Xin Wang, Yuqi Xing, Xin Lü

**Affiliations:** ^1^College of Food Science and Engineering, Henan University of Technology, Zhengzhou, China; ^2^College of Food Science and Engineering, Northwest A&F University, Yangling, China; ^3^Science Center for Future Foods, Jiangnan University, Wuxi, China

**Keywords:** lignin degradation, multi-copper polyphenol oxidoreductase, characterization, enzyme, cleavage mechanism

## Abstract

Identifying the enzymes involved in lignin degradation by bacteria is important in studying lignin valorization to produce renewable chemical products. In this paper, the catalytic oxidation of lignin by a novel multi-copper polyphenol oxidoreductase (OhLac) from the lignin degrader *Ochrobactrum* sp. J10 was explored. Following its expression, reconstitution, and purification, a recombinant enzyme OhLac was obtained. The OhLac enzyme was characterized kinetically against a range of substrates, including ABTS, guaiacol, and 2,6-DMP. Moreover, the effects of pH, temperature, and Cu^2+^ on OhLac activity and stability were determined. Gas chromatography-mass spectrometer (GC-MS) results indicated that the β-aryl ether lignin model compound guaiacylglycerol-β-guaiacyl ether (GGE) was oxidized by OhLac to generate guaiacol and vanillic acid. Molecular docking analysis of GGE and OhLac was then used to examine the significant amino residues and hydrogen bonding sites in the substrate–enzyme interaction. Altogether, we were able to investigate the mechanisms involved in lignin degradation. The breakdown of the lignocellulose materials wheat straw, corn stalk, and switchgrass by the recombinant OhLac was observed over 3 days, and the degradation results revealed that OhLac plays a key role in lignin degradation.

## Introduction

The natural constituents of plant biomass are cellulose, lignin, hemicellulose, pectin, and so on. Among these, the aromatic heteropolymer lignin, which forms a natural physical barrier to the hydrolysis of cellulose, poses a great challenge in the utilization of lignocellulosic biomass (Yang and Lü, 2021). Many commercial chemical and physical methods aimed at lignin degradation are non-specific, energy consuming, and harmful to the environment. Biological methods involving microbial enzymes to oxidize and break down lignin structures have been studied extensively. Ligninolytic enzymes such as lignin peroxidases, manganese-dependent peroxidases, laccases, and dye-decolorizing peroxidases are effective in lignin degradation (Zhu et al., 2020; Kuppuraj et al., 2021).

The multi-copper oxidase group forming part of a large class of enzymes (Janusz et al., 2017), especially laccases (benzenediol: oxygen oxidoreducutases; EC 1.10.3.2), are known as one of the important enzymes of wood-destroying microorganisms. Notably, these enzymes can oxidize substrates by four redox-active copper ions as the cofactors and produce only water as a by-product; therefore, they are termed “green enzymes” (Riva, 2006). Laccases from fungi, bacteria, plants, and insects vary greatly in structure, molecular weight, or oligomeric state (Mayer and Staples, 2002; Navaneetha et al., 2011; Mate and Alcalde, 2015). Most characterization of fungi laccases has been conducted in *Trametes versicolor* and *Pycnoporus cinnabarinus*, where they have been shown to play important roles in lignin degradation (Youn et al., 1995; Eggert et al., 1997). Moreover, laccases have been shown to be abundant in bacteria, such as *Bacillus pumilus*, *Bacillus licheniformis*, and *Escherichia coli*, in which they are active against a wide range of substrates (Hullo et al., 2001; Xu et al., 2007; Chang et al., 2014). Traditional laccases were previously reported to consist of three structural domains and contained four copper ions in the active site: T1 Cu was responsible for the characteristic blue color and caused oxidation of substrate molecules by one-electron abstraction (Jones and Solomon, 2015); T2 Cu and a pair of T3 Cu formed a trinuclear cluster in which molecular oxygen was reduced to water (Mate and Alcalde, 2015).

Recently, laccases from bacteria that lack one of the three structural domains were identified and designated as “small laccases” because of their sequence similarity but smaller size compared with traditional laccases (Machczynski et al., 2004; Majumdar et al., 2014). Small laccases, such as SLAC from *Streptomyces coelicolor* and Ssl1 from *Streptomyces sviceus*, were extensively characterized and reported to be effective lignin-degrading enzymes (Gunne et al., 2014; Majumdar et al., 2014). Furthermore, these enzymes were demonstrated to have high oxidizing ability, thermal stability, and pH versatility. A novel polyphenol oxidase, P-PPO, with a lower molecular weight (33.4 kDa) was found and amplified from *Paenibacillus* sp., but unfortunately no activity was found with all the substrates tested against this enzyme (Granjatravez et al., 2018). Therefore, polyphenol oxidases from different kinds of bacteria require further investigation. In addition, detailed information about the lignin-degrading mechanism of these enzymes has rarely been studied using lignin model compounds and raw lignocellulosic biomass as substrates.

In the present study, a novel multi-copper polyphenol oxidoreductase, OhLac, was amplified and identified from the lignin-degrading bacteria *Ochrobactrum* sp. J10, which was isolated from rotten wood in Qinling, China, and reported in our previous study (Yang et al., 2017). The enzyme activity and biochemical characterization of OhLac were studied using a broad range of substrates. Furthermore, the lignin cleavage mechanism of OhLac was explored and assessed in the context of its lignin-degrading abilities.

## Materials and Methods

### Strains, Plasmids, and Reagents

The lignin-degrading bacteria *Ochrobactrum* sp. J10 (KX822680), which provided a source of lignin peroxidase, were isolated previously from rotten wood in Qinling Mountain, China (Yang et al., 2017). *E. coli* DH5α, BL21(DE3; TransGen Biotech Co., Ltd), and the plasmid pET-28a(+) (Takara Bio, Otsu, Japan) were used for cloning and expression experiments. The genomic DNA was extracted using an Ezup column bacteria genomic DNA purification kit (Shanghai Sangon Biotech Co., Ltd.). The Ni-NTA His Bind resin column (Novagen, Germany) was used to purify the enzyme. 2,2’-Azino-bis(3-ethylbenzothiazoline-6-sulfonic acid; ABTS), guaiacol, 2,6-dimethoxyphenol (2,6-DMP), and [N,O-bis (trimethylsilyl) trifluoroacetamide and (trimethylchlorosilane)] (BSTFA+TMCS) were purchased from Sigma Aldrich (St. Louis, MO, United States). Guaiacylglycerol-β-guaiacyl ether (GGE; Tokyo Chemical Industry Co., Ltd.), as the lignin model compound, was used to study the degradation mechanism of the enzyme.

### Cloning, Overexpression, and Purification of Recombinant OhLac

The genomic DNA of *Ochrobactrum* sp. J10 was extracted using a genomic DNA purification kit after the cells were cultured and centrifuged. The gene encoding multi-copper polyphenol oxidoreductase from *Ochrobactrum* sp. J10 (OhLac) was amplified using primers, which were designed according to the gene sequence of the hypothetical laccase: ML11-F: 5′-CATG*CCATGG*GCATGAATACATATCACCCATTCAGTCTT-3′, ML11-R: 5′-AACG*CTCGAG*TGCCTCCTTCATTCCGATAA AG-3′ (the restriction sites *Nco*I and *Xho*I were underlined). The OhLac gene was cloned into an expression vector pET28a(+) and a recombinant engineered bacteria containing OhLac was constructed. Then, the recombinant enzyme was expressed with an N-terminal His6 fusion protein in *E. coli* BL21(DE3) after induction with 0.2 mM IPTG (Camarero et al., 2005; Yang et al., 2017). After cell collection and disruption, the cell lysates were loaded onto a Ni-NTA His Bind resin column and the recombinant OhLac protein was eluted with elution buffer (20 mM Tris–HCl, 150 mM NaCl, and 250 mM imidazole, pH 8.0). SDS-PAGE was then performed to analyze the OhLac protein. Purified OhLac was dialyzed against 20 mM Tris–HCl at pH 8.0. The protein concentration was determined using the BCA method with bovine serum albumin as the standard (Ye et al., 2012; Bu et al., 2013).

### Enzymatic Assays

All enzyme assays were carried out using a microplate reader (Victor X3, PerkinElmer, United States). The oxidation of ABTS, guaiacol, and 2,6-DMP was performed at 420 nm (ε_420_ = 36,000 M^–1^ cm^–1^), 470 nm (ε_470_ = 26,600 M^–1^ cm^–1^), and 468 nm (ε_468_ = 49,600 M^–1^ cm^–1^), respectively. One unit of enzyme activity (U) was defined as the amount of enzyme required to oxidize 1 μmol of substrate per minute.

### Effect of pH, Temperature, and Cu^2+^ on OhLac Activity and Stability

The optimum pH for OhLac activity was determined using ABTS, guaiacol, and 2,6-DMP as the substrates at different pH (2.0–12.0) at 30°C. Purified OhLac was incubated in 100 mM citric acid-disodium hydrogen phosphate buffer (pH 2.0–7.0), 100 mM Tris–HCl buffer (pH 8.0–9.0), and 100 mM glycine-NaOH buffer (pH 10.0–12.0) to oxidize the substrates. The effect of temperature was determined by performing assays at temperatures ranging from 20 to 90°C at the optimum pH determined previously, ABTS (pH 3.0), guaiacol (pH 5.0), and 2,6-DMP (pH 5.0). The relative activity for each substrate was calculated considering the pH and temperature of maximum activity as 100%. To determine the enzyme stability at different pH values and temperatures, OhLac was incubated at every pH and temperature condition for 1 h, then the residual activity was measured. The enzyme activity at 0 h was defined as 100%. The effect of Cu^2+^ on OhLac activity was assayed by adding different concentrations of CuSO_4_ (0.25, 0.50, 1.00, 2.50, 5.00, 7.50, and 10.00 mM) into various substrate systems at the optimum pH and temperature (ABTS: pH 3.0, 50°C; guaiacol: pH 5.0, 40°C; 2,6-DMP: pH 5.0, 30°C).

### Steady-State Kinetic Assays of OhLac

Steady-state kinetic parameters were measured by non-linear curve fitting to the obtained enzyme activities (using GraphPad Prism 5 software) and were fit according to the Michaelis–Menten equation (Rahmanpour and Bugg, 2015; Brissos et al., 2017; Sugawara et al., 2017). The catalytic constants (*V*_max_, *K*_m_, *k*_cat_, and *k*_cat_/*K*_m_) were determined by incubating OhLac with various concentrations of substrates, including ABTS (0.1, 0.2, 0.5, 1.0, 3.0, 5.0, 7.0, 10.0, 15.0, and 20.0 mM), guaiacol (0.1, 0.3, 0.5, 1.0, 1.5, 2.0, 2.5, 3.0, 3.5, 4.0, 4.5, and 5.0 mM), and 2,6-DMP (0.1, 0.3, 0.5, 0.7, 1.0, 2.0, 3.0, 4.0, and 5.0 mM). OhLac activity comparison was made with the commercial fungal laccase from *T. versicolor* (TvL; CAS: 80498-15-3) purchased from Sigma Aldrich (St. Louis, MO, United States). The steady-state kinetic parameters were determined and calculated using ABTS, guaiacol, and 2,6-DMP as substrates at the optimum pH, temperature, and Cu^2+^ concentration (ABTS: pH 3.0, 50°C, Cu^2+^ 0.25 mM; guaiacol: pH 5.0, 40°C. Cu^2+^ 0.50 mM; 2,6-DMP: pH 5.0, 30°C, Cu^2+^ 5.00 mM).

### Degradation of β-aryl Ether Lignin Model Compound by OhLac

The β-aryl ether dimer GGE was used as the lignin model compound to study the lignin-degrading mechanism of OhLac. The GGE was dissolved in methanol at a concentration of 10 mM. The enzymatic reaction systems consisted of 2 mM GGE, 0.1 mg/ml OhLac, 0.25 mM CuSO_4_, 1 mM ABTS, and 20 mM ammonium bicarbonate buffer. The reactions were carried out at 40°C for 4 h. Then, the products of GGE degradation were analyzed by gas chromatography-mass spectrometer (GCMS)-QP2010 Ultra (Shimadzu, Kyoto, Japan). After extraction by ethyl acetate and derivatization by BSTFA+TMCS, the samples were injected into a GC-MS system to separate and analyze the compounds. The sample treatment method and operating parameters used were similar to those defined in our previous description (Yang et al., 2018).

### Molecular Docking of the Protein Ligand

To provide deep insight into the binding of the enzyme OhLac and the substrate GGE, AutoDock 4.2, AutoGrid 4.2, and PyMOL were used to perform and set up docking calculations. The 3D structure of OhLac was predicted by I-TASSER. The structure file of GGE was prepared using ZINC database^1^ and AutoDock Tools. Polar hydrogen atoms were added, and the formats of the protein receptor and ligand were converted from PDB to PDBQT using AutoDock Tools. The docking of OhLac-GGE was carried out by AutoDock 4.2 with a Local Search Parameters algorithm method to define the docking site on protein target. The center of the docking box was set as the putative catalytic active center (75 × 56 × 61) in a grid with 40-Å spacing. The other parameters were set to the default values. The best conformation according to the scoring function was chosen as that with the lowest Gibbs energy after the docking search was completed. Each docking experiment was performed for 50 runs in per ligand structure; then, the best pose was saved. In addition, the interactions of OhLac-GGE, such as hydrogen bonds, were explored using PyMOL software (Lu et al., 2017; Xie et al., 2019).

### Lignin Degradation of the Native Lignocellulose Material by OhLac

Three kinds of raw lignocellulosic materials (wheat straw, corn stalk, and switchgrass) were selected as the substrates for OhLac to degrade. After being crushed and passed through a 40-mesh sieve, the three materials were added at the ratio of 15% (w/v) to the enzymatic reactions, which consisted of 0.25 mM CuSO_4_, 1.0 mM ABTS, 1.0 mM H_2_O_2_, and 20 mg/ml OhLac (pH 6.0), respectively. The resulting solutions were incubated at 40°C for 3 days. The lignin degradation ratio was determined using the Klason lignin method described previously (Yang et al., 2018).

### Statistical Analysis

All experiments were performed in triplicate, and the results were expressed as mean ± standard deviation. Data analysis was performed by ANOVA and Duncan test using SPSS 16.0. In all analysis, *p* < 0.05 was considered significant.

## Results

### Bioinformatic Analysis of OhLac in *Ochrobactrum* sp. J10

**FIGURE 1:**
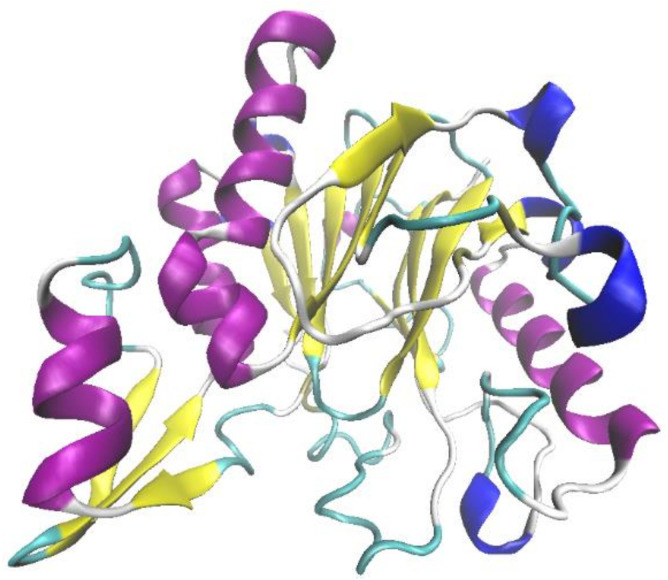
The prediction on tertiary structure of OhLac using Swiss-Model.

*Ochrobactrum* sp. J10 was revealed to exhibit potentially high lignin-degrading activity in our previous study (Yang et al., 2017). Following an NCBI search and PCR amplification, the gene *OhLac*, encoding the OhLac enzyme in *Ochrobactrum* sp. J10, was amplified from chromosomal DNA. Analysis of the nucleotide sequence of *OhLac* showed that it contained a single open reading frame of 837 bp, which encoded a protein monomer of 279 amino acids with a deduced theoretical pI of 5.98. The cloned enzyme was found to be a Multi-copper Polyphenol Oxidoreductase (IPR003730) after analysis of protein domain databases. The sequence of *OhLac* (Supplementary Figure 1) was used to perform a BLAST search in UniProt^2^, and the result showed that it was similar to the putative polyphenol oxidase YlmD (ID O31726) in *Bacillus subtilis* 168, with 98.92% sequence identity; however, there was no report of YlmD having lignin-degrading potential. The 3D structure of OhLac was modeled using Swiss-Model according to the obtained sequences (Figure 1). The structure of OhLac was identified with 48.33% identity with the template protein (PDB ID 1t8h.1.A) from *Geobacillus stearothermophilus*, including the central β-sheets and peripheral α-helices.

**FIGURE 2:**
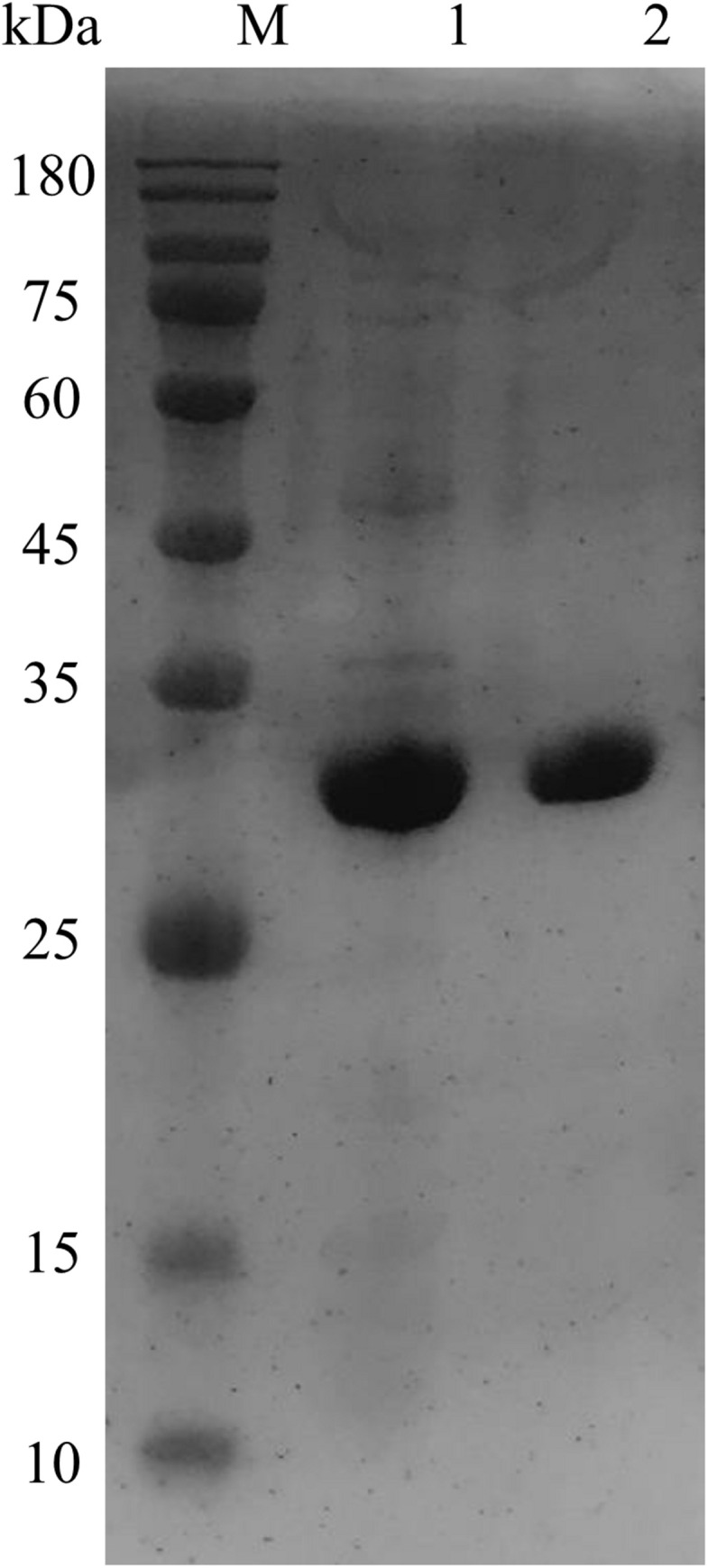
The SDS-PAGE results of purified OhLac using Ni-NTA column. Lane 1: crude enzyme, lane 2: purified OhLac, and M: maker.

The transformation of *OhLac* was carried out successfully, and the expression of the OhLac enzyme followed induction by IPTG, which was added to the culture medium. The recombinant protein was purified using a Ni-NTA column, and the molecular weight of purified OhLac is shown in Figure 2. The protein content of crude extract was 281.90 mg/L. After purification, the protein content of purified OhLac was 76.20 mg/L. The OhLac had a specific activity of 177.03 U/mg when ABTS is used as the substrate. The recovery rate was 35.27% (in Supplementary Table 1).

### Effects of pH, Temperature, and Cu^2+^ on OhLac Activity and Stability

The effects of pH, temperature, and Cu^2+^ on OhLac activity and stability were examined with different substrates, including ABTS, guaiacol, and 2,6-DMP.

**FIGURE 3:**
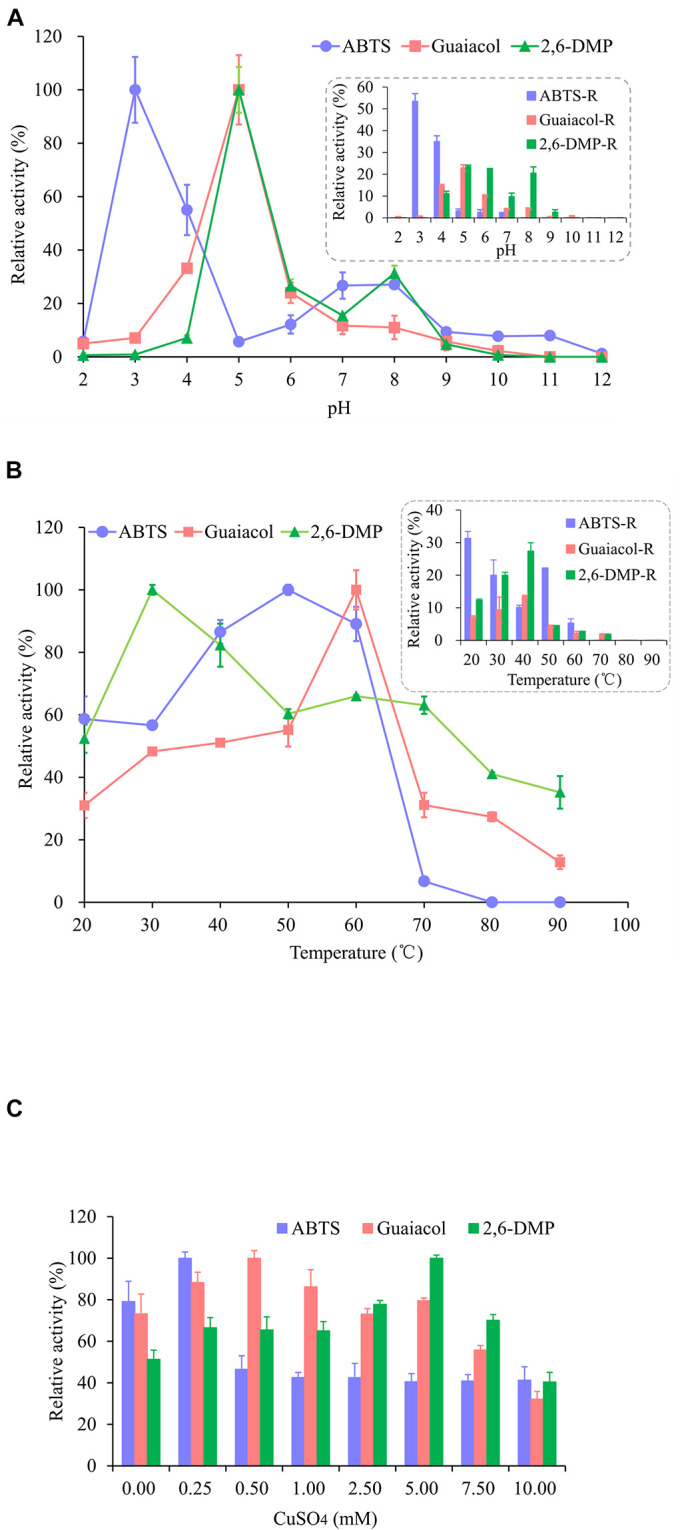
Effects of pH **(A)**, temperature **(B)**, and Cu^2+^ **(C)** on the activity and the stability of recombinant OhLac. **(A)** The relative activity of OhLac at different pH (2.0–12.0) was determined using ABTS, guaiacol, and 2,6-DMP as the substrates. And the residual relative activity (ABTS-R, Guaiacol-R, and 2,6-DMP-R) of OhLac was measured after 1 h at the corresponding pH. **(B)** The relative activity of OhLac at different temperature (20–90°C) was determined using ABTS, guaiacol, and 2,6-DMP as the substrates. And the residual activity of OhLac was measured after 1 h at different temperature. **(C)** The effect of Cu^2+^ on relative activity of OhLac adding different concentrations of CuSO4 (0.25, 0.50, 1.00, 2.50, 5.00, 7.50, and 10.00 mM). The relative activity for each substrates was calculated considering the maximum activity as 100% at different pH, temperature and CuSO_4_ concentrations, respectively.

As shown in Figure 3A, the enzyme activity of OhLac was determined at pH values ranging from 2.0 to 12.0 and these values were shown to have a great influence on the enzyme activity of the recombinant OhLac enzyme. Overall, the optimal pH range for OhLac activity was 3.0–5.0, which were acidic conditions. When the pH was greater than 7.0, the enzyme activity against all substrates reduced, and the optimal pH for enzyme activity was affected by the different substrates. With ABTS as the substrate, the optimum pH was 3.0. However, when the pH was 2.0, there was almost no enzyme activity with ABTS, whereas, when the pH was 4.0, the relative enzyme activity was reduced by half. The optimal pH was shown to be 5.0 using guaiacol and 2,6-DMP, both aromatic compounds, as substrates. According to the results of an enzyme activity assay at different pH values, the relative enzyme activity remained at 53.4% at pH 3.0 after 1 h with ABTS. However, the enzyme activity stability of OhLac when using guaiacol and 2,6-DMP (22.85 and 23.47%, respectively) was lower. The optimal pH of bacterial laccase has been reported to range from 3.0 to 9.0 (Prins et al., 2015; Liu et al., 2020; Yang et al., 2020), varying according to the strains and substrates, while most fungal laccases are suitable for catalyzing under acidic conditions (Baldrian, 2006). The laccase OcCueO from *Ochrobactrum* sp., with a molecular weight of 55.1 kDa, exhibited maximum enzyme activity at pH 4.0 and 8.0 using ABTS as the substrate (Granjatravez et al., 2018).

Raising the reaction temperature is beneficial to increasing enzyme activity, but too high a temperature may cause the enzyme protein to denature and even lose activity. So, the effect of temperature on OhLac activity was studied, and the results are shown in Figure 3B. The enzyme activity of OhLac was shown to increase at first and then decrease with increasing temperature. Using the three substrates, the relative enzyme activity was higher at 30–60°C. When the temperature exceeded 70°C, the enzyme activity dropped sharply or activity was lost. According to reports, most laccases operate under an optimum catalytic temperature of 40–70°C. However, some laccases from thermophilic microorganisms (such as *Thermus thermophilus* and *Trametes pubescens*) can withstand a temperature of 90°C (Singh et al., 2009; Zheng et al., 2012; Jing et al., 2013).

Different concentrations of Cu^2+^ were added to detect the impact of Cu^2+^ on the enzyme activity of OhLac. As shown in Figure 3C, with an increasing Cu^2+^ concentration, the relative enzyme activity increased first and then decreased. Interestingly, it was found that there was a certain amount of enzyme activity observed when CuSO_4_ was not added (0.00 mM), which indicated that the recombinant enzyme OhLac was not Cu^2+^-dependent. Similarly, it was reported that OcCueO, a laccase from *Ochrobactrum* sp., showed enzyme activity without the addition of Cu^2+^ (Granjatravez et al., 2018). In this study, compared with the control sample without Cu^2+^, the samples containing significant concentrations of Cu^2+^ showed higher activity. For example, OhLac activity increased by about 10% with the addition of 0.25 mM CuSO_4_ using ABTS as the substrate, but when the concentration of CuSO_4_ was more than 0.25 mM, the enzyme activity decreased by about 40%, which was significant. It was therefore suggested that the excess Cu^2+^ might inhibit the enzyme activity of OhLac. Similarly, when guaiacol and 2,6-DMP were used as the substrates, the optimum concentrations of CuSO_4_ added were 0.50 and 5.00 mM, respectively.

### Kinetic Characterization of OhLac

**FIGURE 4:**
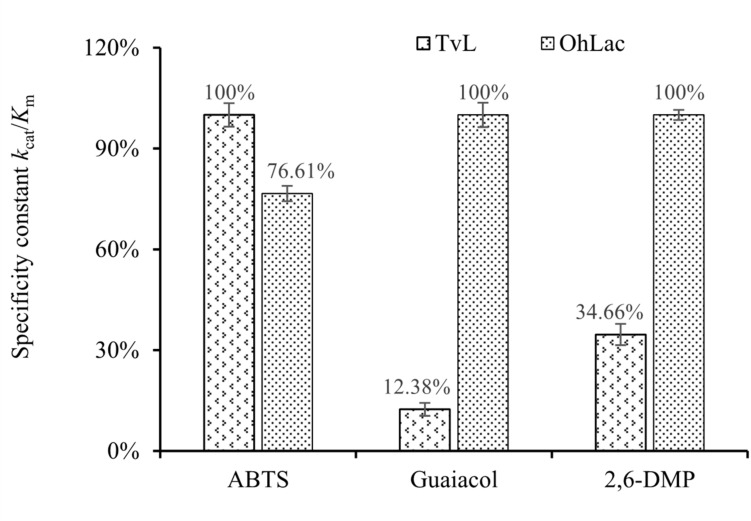
Relative values of specificity constant *k*_*cat*_*/Km* with different substrates catalyzed by fungal laccase TvL and bacterial OhLac.

To characterize the catalytic specificity of recombinant OhLac, three substrates were tested, including ABTS, guaiacol, and 2,6-DMP, using UV-visible spectrophotometric assays. The results showed (in Table 1 and Supplementary Figure 2) that OhLac was active with all substrates. The steady-state kinetic parameters of other multi-copper polyphenol oxidoreductases reported are summarized in Table 2. In comparison, the *V*_max_ value of OhLac was about 60 U/mg, which was much higher than that of other similar enzymes. Moreover, OhLac exhibited *k*_cat_ values of 20–35 s^–1^ for three substrates, but a lower *K*_*m*_ of (523.18 ± 9.20) μM for guaiacol, giving a *k*_cat_/*K*_m_ value of 6.47 × 10^4^ M^–1^ s^–1^ for this substrate. Interestingly, the *k*_cat_/*K*_m_ value of OhLac was lower than that of CotAs with higher Mw (about 65 kDa) from *Bacillus* sp. (Koschorreck et al., 2008; Li et al., 2018), but the specificity of OhLac was much higher than most other multi-copper oxidoreductases, especially a smaller laccase with a similar Mw (about 32 kDa) from *Streptomyces* sp. In addition, the activity of OhLac was compared with that of the commercially available fungal laccase from *Trametes versicolor* (TvL). As shown in Figure 4, the specific activity of OhLac toward ABTS was 76.61% of that measured for TvL. However, the specific activity of OhLac was three- to eight-fold higher than that of TvL when the aromatic compounds, guaiacol and 2,6-DMP, were used as substrates.

**TABLE 1:** The kinetic parameters of recombinant OhLac.

Substrates	*V*_*max*_ (U/mg)	*K*_*m*_ (μM)	*k*_*cat*_ (s^–1^)	*k*_*cat*_/*K*_*m*_ (M^–1^ s^–1^)
ABTS	68.49 ± 6.85	1,061.64 ± 9.78	35.02 ± 3.26	3.30 × 10^4^
Guaiacol	66.23 ± 5.76	523.18 ± 9.20	33.86 ± 5.59	6.47 × 10^4^
2,6-DMP	41.67 ± 5.41	1,125.01 ± 5.12	21.30 ± 3.11	1.89 × 10^4^

*The data were presented as the mean ± SD (*n* = 3).*

**TABLE 2:** The review of steady-state kinetic parameters of multi-copper polyphenol oxidoreductases.

Enzyme	Species	Mw (kDa)	ABTS					2,6-DMP				References
			*V*_*max*_ (U/mg)	*K*_*m*_ (μM)	*k*_*cat*_ (s^–1^)	*k*_*cat*_/*K*_*m*_ (M^–1^ s^–1^)		*V*_*max*_ (U/mg)	*K*_*m*_ (μM)	*k*_*cat*_ (s^–1^)	*k*_*cat*_/*K*_*m*_ (M^–1^ s^–1^)	
OhLac	*Ochrobactrum* sp. J10	31	68.49 ± 6.85	1,061.64 ± 9.78	35.02 ± 3.26	3.30 × 10^4^		41.67 ± 5.41	1,125.01 ± 5.12	21.30 ± 3.11	1.89 × 10^4^	This paper
CcCueO	*Ochrobactrum* sp.	55.1	1	1,463	0.93	635.80		0.90	0.31	0.90	2,929.90	Granjatravez et al., 2018
CotA	*Bacillus subtilis*	65	–	104 ± 5	26.80 ± 2.00	2.60 × 10^5^		–	–	–	–	Li et al., 2018
CotA	*Bacillus altitudinis* SYBC hb4	58.9	51.45	88	50.30	5.70 × 10^5^		–	–	–	–	Zhang et al., 2016
CotA	*Bacillus licheniformis*	65	–	6.50 ± 0.20	83	–		–	56.70 ± 1.00	28	–	Koschorreck et al., 2008
LMCOs	*Bacillus coagulans*	59.7	–	31	69 ± 3.10	–		–	0.63	17 ± 0.80	–	Ihssen et al., 2015
LMCOs	*Bacillus clausii*	58.4	–	132	90 ± 2.30	–		–	8.54	65 ± 15.20	–	Ihssen et al., 2015
LCC3	*Trametes trogii* BAFC 463	58	–	250 ± 9	399 ± 20 seg^–1^	–		–	2,095 ± 63	329 ± 32 seg^–1^	–	Campos et al., 2016
Lac21	*Ahrensia* sp. R2A130	50	NA	NA	NA	NA		–	216	17.83	8.25 × 10^4^	Fang et al., 2012
P-PPO	*Paenibacillus* sp.	33.4	NA	NA	NA	NA		NA	NA	NA	NA	Granjatravez et al., 2018
TtSLAC	*Thermus thermophilus* HJ6	26.3	–	490	1.48	3.02 × 10^3^		–	110	2.93	2.66 × 10^4^	Kim et al., 2015
Ssl1	*Streptomyces sviceus*	35.8	–	–	–	–		–	890	0.32	361.42	Gunne et al., 2014
SLAC	*Streptomyces coelicolor* (SCO6712)	32.7	–	(0.80 ± 0.10) × 10^3^	7.70 ± 0.30	(10 ± 1) × 10^3^	–	–	(1.10 ± 0.20) × 10^3^	4.00 ± 0.20	(3.50 ± 0.60) × 10^3^	Sherif et al., 2013
SLAC	*Streptomyces coelicolor* A3(2)	35.5	3.10 ± 0.03	(5.89 ± 1.69) × 10^3^	9.50 ± 1.43	(1.61 ± 0.44) × 10^3^		7.07 ± 0.32	(5.09 ± 1.34) × 10^3^	8.22 ± 1.03	(1.62 ± 0.39) × 10^3^	Prins et al., 2015
SLAC	*Streptomyces coelicolor*	37.7	–	–	–	–		–	2,850	0.87	3.05 × 10^2^	Toscano et al., 2013
Ssl1	*Streptomyces sviceus*	32.5	–	360	7.38	2.05 × 10^4^		–	890	0.32	–	Gunne and Urlacher, 2012
SvSL	*Streptomyces viridochromogenes*	39	–	(0.30 ± 0.01) × 10^3^	8.00 ± 0.15	2.66 × 10^4^		–	(4.50 ± 0.30) × 10^3^	1.90 ± 0.12	420	Trubitsina et al., 2015

*NA: no activity observed.*

*–: not determined.*

### Oxidation of β-aryl Ether Lignin Model Compound by OhLac

**FIGURE 5:**
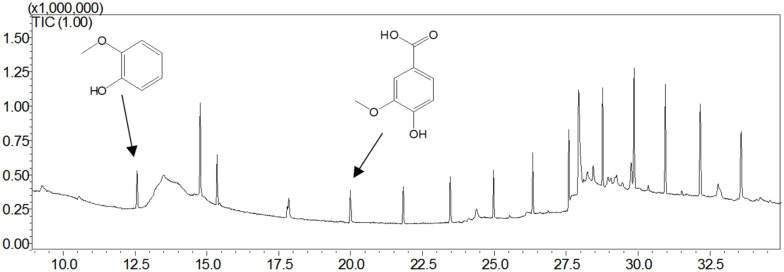
The products analysis of lignin model compound GGE degraded by recombinant OhLac.

Guaiacylglycerol-β-guaiacyl ether was used to study the action of lignin-oxidizing enzymes. To explore the cleavage of lignin substrates, the reaction products of GGE degraded by OhLac were determined using GC-MS. The ABTS was added as a mediator and the results are shown in Figure 5. Using the NIST library, some of the aromatic compounds, aldehydes, and acids were analyzed according to their ion chromatogram results. In particular, guaiacol (RT = 12.650 min) and vanillic acid (RT = 20.342 min), aromatic monomer compounds, were identified as reaction products.

### The Analysis of Enzyme–Substrate Interactions and Prediction of Binding Sites

**FIGURE 6:**
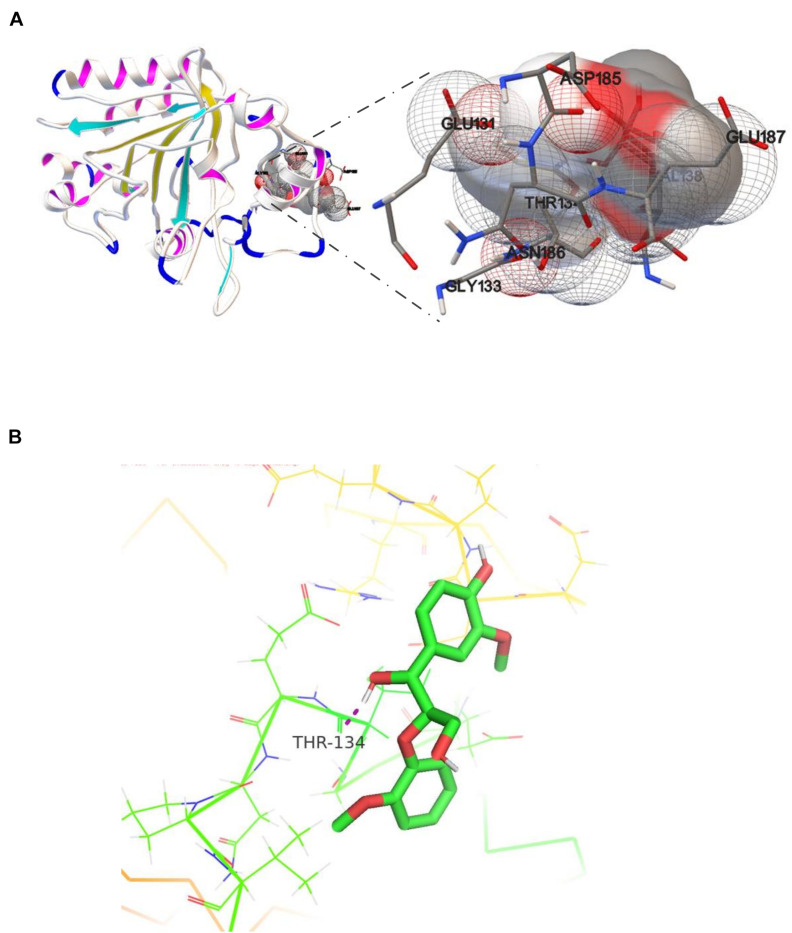
**(A)** The molecular docking result of recombinant OhLac and GGE, **(B)** The hydrogen bonding between GGE and OhLac.

Studies of small-molecule compounds docking in the binding sites of receptors revealing the interactions in the complexes are important to analyzing the catalytic sites of the enzyme–substrates. The results from the analysis of OhLac-GGE docking at the lowest binding energy are shown in Figure 6A. Seven amino residues in OhLac, namely, GLU131, GLY133, THR134, VAL138, ASP185, ASN186, and GLU187, interacted with the substrate GGE directly, which indicated that these seven amino residues could be very significant sites in the catalytic process. Hydrogen bonding between OhLac and GGE was analyzed using PyMOL, and the results are shown in Figure 6B.

### Oxidation and Degradation of Raw Lignocellulose Materials by OhLac

**FIGURE 7:**
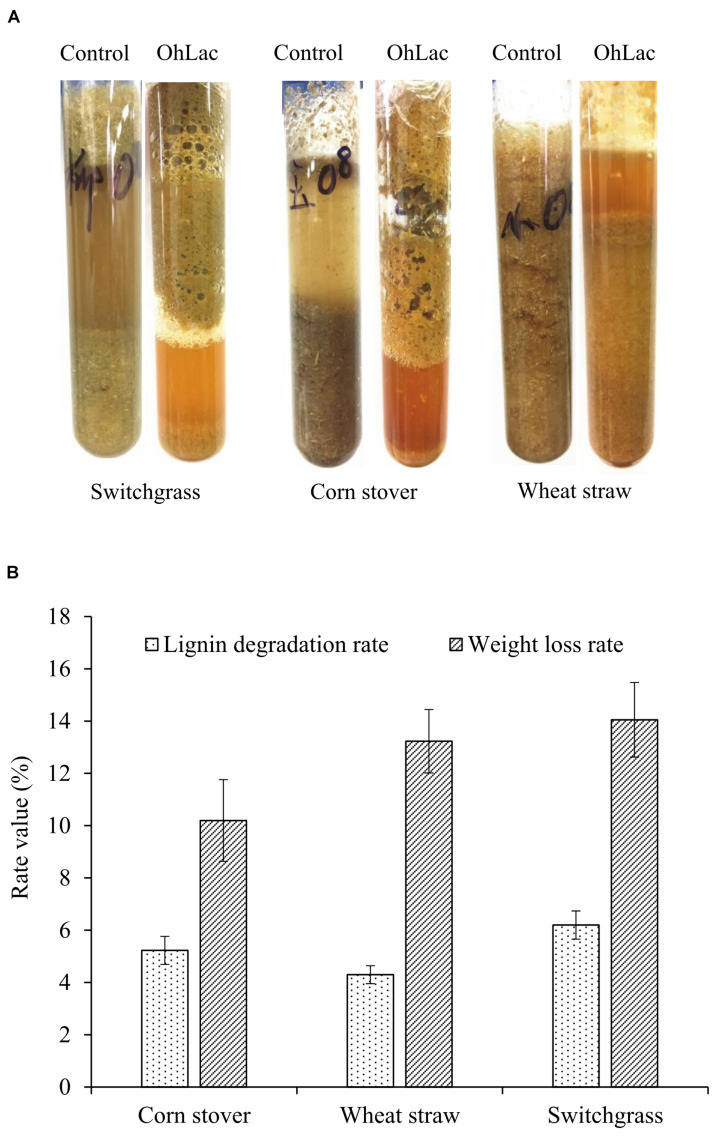
The degradation of lignocellulose material by recombinant OhLac. **(A)** The appearance changes after degradation by OhLac. **(B)** The determination results of the lignin degradation rate and weight loss rate degraded by OhLac.

To verify the oxidation activity of the recombinant OhLac against native lignin, powdered lignocellulose materials, including switchgrass, corn stalk, and wheat straw, were used. Moreover, the visible changes in the native materials were observed. The results of degradation by OhLac for 24 h are shown in Figure 7A. Following treatment, the appearance of the raw materials had changed significantly. For example, the particle size of the lignocellulosic material was reduced, and the surface changed from smooth to fluffy, causing some of the particles to float on the surface of degradation liquid. Simultaneously, compared with that of the control samples, the enzymatic hydrolysate was darkened and yellowish-brown after the degradation. These results might be due to the dissolution of internal substances, such as proteins, pigments, polysaccharides, or others, after the structural depolymerization of the raw materials by OhLac. The above phenomena were confirmed by the determination of the lignin degradation rate and weight loss rate (Figure 7B). After 3 days of degradation, the weight loss rate for the three materials was 10–15%. The lignin degradation rate was highest for switchgrass (∼6.20%), followed by corn stalk (5.2%), and wheat straw was the worst (4.30%). Different kinds of plant biomass exhibit differences in their structural composition and properties, such as the content of β-O-4 bonds, methoxy content, or condensation, which leads to a difference in degradation rates (Mann et al., 2009; Santos et al., 2012).

## Discussion

The polyphenol oxidoreductase OhLac from *Ochrobactrum* sp. J10 was demonstrated to be a potential lignin-degrading enzyme. The results of the kinetic characterization indicated that OhLac might show better activity and potential for certain lignin-degrading applications. It was well known that the traditional fungal and bacterial laccases contained three domains and four copper atoms in their active center, whose molecular weights were about 60 kDa, such as CotA (Koschorreck et al., 2008). In addition, another form of the polyphenol oxidoreductase was found with two domains (about 30 kDa) in bacteria or called “small laccases” (Majumdar et al., 2014). The property of small laccases was reported to be markedly different from that of traditional laccases. It had been proved that they had activities against a wide range of phenolic compounds (Prins et al., 2015). Besides, they had the advantages of high oxidizing power, thermal stability, and pH versatility (Lee et al., 2019). However, more detailed biochemical and structural studies of various smaller size laccases should be conducted and explored deeply to promote their application to lignin degradation.

Guaiacylglycerol-β-guaiacyl ether is a typical phenolic model compound containing the β-O-4 bond. It has been reported that the intersubunit content of β-O-4 bonds is more than 50% in lignin (Villaverde et al., 2009). These peaks (guaiacol and vanillic acid) appeared to correspond to the Cα-Cβ oxidative products of GGE when degraded by OhLac. Similarly, OcCueO has been reported to decompose lignosulfonate to form vanillin acid as a product, which indicates cleavage of the Cα-Cβ bond in lignin (Granjatravez et al., 2018). The small laccases from *Streptomyces* (SCLAC) were studied to investigate the degradation of the phenolic β-O-4 lignin model compound (LM-OH), and vanillin was identified as a degradation product because of Cα-Cβ bond cleavage. SCLAC could oxidize the non-phenolic β-O-4 lignin model compound (LM-OMe) to form the corresponding ketone product (Majumdar et al., 2014). We found that there was a hydrogen bond between the substrate GGE and the amino acid THR134 of OhLac. Specifically, the THR134 residue could form a hydrogen bond with the hydroxyl group attached to the Cα position in GGE, which might offer atoms as hydrogen bond donors and acceptors (Yang et al., 2015). The results indicated that these amino residues were very important putative catalytic sites. The contribution of every active site to enzyme activity will be explored in future work.

**FIGURE 8:**
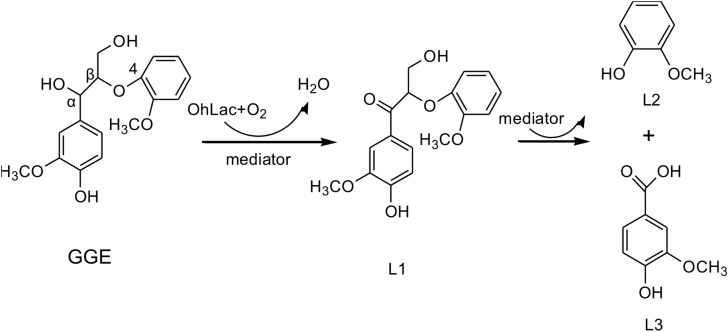
The presumed cleavage process of lignin model compound GGE degraded by OhLac.

The above results from the studies of degradation and docking of lignin model compounds indicated that OhLac was available to oxidize lignin. Therefore, lignin cleavage by some bacteria might be related to the action of multicopper oxidases. Laccase may only be responsible for the radical initiation step, while the downstream Cα-Cβ bond cleavage may be the result of spontaneous reaction (Leonowicz et al., 2001; Majumdar et al., 2014). The oxidation of the lignin model compound by a fungal laccase led to the formation of the corresponding ketone product instead of Cα-Cβ bond cleavage (Li et al., 1999). Laccases catalyze oxidation of lignin, initially producing a phenoxy radical (Perna et al., 2019). It had often been reported that Cα-oxidation, rather than ether bond cleavage, was the main result of laccase-catalyzed lignin degradation system (Heap et al., 2014; Hilgers et al., 2019). A non-phenolic lignin model, veratrylglycerol-β-guaiacyl ether (VBG), was used to explore the degradation of lignin by a laccase/HBT system. The results showed that the Cα-ketone analog of VBG (VBGox) and two ether cleavage products were identified, which indicated Cα-oxidation cleavage (Hilgers et al., 2020). In this study, a mixture of oxidative degradation products was formed, which were not fully characterized. However, the products were identified and characterized as guaiacol and vanillic acid. Therefore, it was supposed that during the oxidation of lignin by OhLac, oxidation of the α-carbon center leads to the formation of the ketone product, and then continued oxidation causes the cleavage of the Cα-Cβ bond of lignin (Figure 8). OhLac is thought to breakdown dimeric lignin GGE by cleaving the Cα-Cβ bond and to produce guaiacol (L2) and vanillic acid (L3).

## Conclusion

In this paper, a novel bacterial multicopper oxidase, OhLac, from *Ochrobactrum* sp. J10 was identified and characterized. After heterologous expression and purification, we noted that OhLac differed from other multicopper oxidases reported previously. In particular, it had a lower molecular weight and two structural domains. Notably, the results proved that this small multicopper oxidase has the potential to be useful in the oxidation and degradation of lignin, playing a key role in the process. Moreover, the cleavage of the β-O-4 linkage in lignin was explored and discussed as part of the oxidative process. Improved knowledge of bacterial enzymes and their roles in lignin degradation will have a significant impact on a wide array of biotechnologies focused on lignin degradation.

## Data Availability Statement

The original contributions presented in the study are included in the article/Supplementary Material; further inquiries can be directed to the corresponding author/s.

## Author Contributions

CY, LM, XW, and XL conceived and designed the project. CY participated in the majority of experiments, drafted the manuscript and all authors participated in the data analysis and revision of manuscript. LM cloned and expressed the genes. CY and YX completed the purification of recombinant protein. CY, XW, and XL performed the analysis of degradation mechanism. All authors contributed to manuscript preparation and they all read and approved the final manuscript.

## Conflict of Interest

The authors declare that the research was conducted in the absence of any commercial or financial relationships that could be construed as a potential conflict of interest.

## Publisher’s Note

All claims expressed in this article are solely those of the authors and do not necessarily represent those of their affiliated organizations, or those of the publisher, the editors and the reviewers. Any product that may be evaluated in this article, or claim that may be made by its manufacturer, is not guaranteed or endorsed by the publisher.

## Footnotes

1
http://zinc.docking.org/

2
https://www.uniprot.org/

## Abbreviations

OhLac: the multi-copper polyphenol oxidoreductase from lignin degrader *Ochrobactrum* sp. J10
ABTS: 2,2’-azino-bis(3-ethylbenzothiazoline-6-sulfonic acid)
2,6-DMP: 2,6-dimethoxyphenol
GGE: guaiacylglycerol- β-guaiacyl ether
MCOs: multi-copper oxidase
TvL: fungal laccase from *Trametes versicolor*
BSTFA+TMCS: N,O-bis (trimethylsilyl) trifluoroacetamide and (trimethylchlorosilane).

## References

[B1] Baldrian P. (2006). Fungal laccases-occurrence and properties. *FEMS Microbiol. Rev.* 30 215–242. 10.1111/j.1574-4976.2005.00010.x 16472305

[B2] Brissos V. Tavares D. Sousa A. C. Robalo M. P. Martins L. O. (2017). Engineering a bacterial dyp-type peroxidase for enhanced oxidation of lignin-related phenolics at alkaline pH. *ACS Catal.* 7 3454–3465. 10.1021/acscatal.6b03331

[B3] Bu D. Zhou Y. Tang J. Jing F. Zhang W. (2013). Expression and purification of a novel therapeutic single-chain variable fragment antibody against BNP from inclusion bodies of *Escherichia coli*. *Protein Expr. Purif.* 92 203–207. 10.1016/j.pep.2013.10.002 24128692

[B4] Camarero S. Ibarra D. Martinez M. J. Martinez A. T. (2005). Lignin-derived compounds as efficient laccase mediators for decolorization of different types of recalcitrant dyes. *Appl. Environ. Microbiol.* 71 1775–1784. 10.1128/aem.71.4.1775-1784.2005 15812000 PMC1082544

[B5] Campos P. A. Levin L. N. Wirth S. A. (2016). Heterologous production, characterization and dye decolorization ability of a novel thermostable laccase isoenzyme from *Trametes trogii* BAFC 463. *Process Biochem.* 51 895–903.

[B6] Chang Y. C. Choi D. Takamizawa K. Kikuchi S. (2014). Isolation of *Bacillus* sp. strains capable of decomposing alkali lignin and their application in combination with lactic acid bacteria for enhancing cellulase performance. *Bioresour. Technol.* 152 429–436. 10.1016/j.biortech.2013.11.032 24316485

[B7] Eggert C. Temp U. Eriksson K. E. (1997). Laccase is essential for lignin degradation by the white-rot fungus *Pycnoporus cinnabarinus*. *FEBS Lett.* 407 89–92. 10.1016/s0014-5793(97)00301-3 9141487

[B8] Fang Z. M. Li T. L. Chang F. Zhou P. Fang W. Hong Y. Z. (2012). A new marine bacterial laccase with chloride-enhancing, alkaline-dependent activity and dye decolorization ability. *Bioresour. Technol.* 111 36–41. 10.1016/j.biortech.2012.01.172 22377476

[B9] Granjatravez R. S. Wilkinson R. C. Felix G. P. Squina F. M. Fülöp V. Bugg T. (2018). Structural and functional characterisation of multi-copper oxidase CueO from lignin-degrading bacterium *Ochrobactrum* sp. reveal its activity towards lignin model compounds and lignosulfonate. *FEBS J.* 285 1684–1700. 10.1111/febs.14437 29575798

[B10] Gunne M. Hoppner A. Hagedoorn P. L. Urlacher V. B. (2014). Structural and redox properties of the small laccase Ssl1 from *Streptomyces sviceus*. *FEBS J.* 281 4307–4318. 10.1111/febs.12755 24548692

[B11] Gunne M. Urlacher V. B. (2012). Characterization of the alkaline laccase Ssl1 from *Streptomyces sviceus* with unusual properties discovered by genome mining. *PloS One.* 7:e52360–e52368. 10.1371/journal.pone.0052360 23285009 PMC3527528

[B12] Heap L. Green A. Brown D. van Dongen B. Turner N. (2014). Role of laccase as an enzymatic pretreatment method to improve lignocellulosic saccharification. *Catal. Sci. Technol.* 4 2251–2259. 10.1039/c4cy00046c

[B13] Hilgers R. Twentyman-Jones M. Van Dam A. Gruppen H. Zuilhof H. Kabel M. A. (2019). The impact of lignin sulfonation on its reactivity with laccase and laccase/HBT. *Catal. Sci. Technol.* 9 1535–1542. 10.1039/c9cy00249a

[B14] Hilgers R. Van Dam A. Zuilhof H. Vincken J.-P. Kabel M. A. (2020). Controlling the competition: boosting laccase/HBT-catalyzed cleavage of a β-O-4’ linked lignin model. *ACS Catal.* 10 8650–8659. 10.1021/acscatal.0c02154

[B15] Hullo M.-F. Moszer I. Danchin A. Martin-Verstraete I. (2001). CotA of *Bacillus subtilis* is a copper-dependent laccase. *J. Bacteriol.* 183 5426–5430. 10.1128/jb.183.18.5426-5430.2001 11514528 PMC95427

[B16] Ihssen J. Reiss R. Luchsinger R. Thöny-Meyer L. Richter M. (2015). Biochemical properties and yields of diverse bacterial laccase-like multicopper oxidases expressed in *Escherichia coli*. *Sci. Rep.* 5 1–13. 10.1038/srep10465 PMC4464401 26068013

[B17] Janusz G. Pawlik A. Sulej J. Swiderska-Burek U. Jarosz-Wilkolazka A. Paszczynski A. (2017). Lignin degradation: microorganisms, enzymes involved, genomes analysis and evolution. *FEMS Microbiol. Rev.* 41 941–962. 29088355 10.1093/femsre/fux049 PMC5812493

[B18] Jing S. Feng P. Cui B. (2013). Purification, biochemical characterization and dye decolorization capacity of an alkali-resistant and metal-tolerant laccase from *Trametes pubescens*. *Bioresour. Technol.* 128 49–57. 10.1016/j.biortech.2012.10.085 23196221

[B19] Jones S. M. Solomon E. I. (2015). Electron transfer and reaction mechanism of laccases. *Cell Mol. Life Sci.* 72 869–883. 10.1007/s00018-014-1826-6 25572295 PMC4323859

[B20] Kim H. W. Lee S. Y. Park H. Jeon S. J. (2015). Expression, refolding, and characterization of a small laccase from *Thermus thermophilus* HJ6. *Protein Expr. Purif.* 114 37–43. 10.1016/j.pep.2015.06.004 26073095

[B21] Koschorreck K. Richter S. M. Ene A. B. Roduner E. Schmid R. D. Urlacher V. B. (2008). Cloning and characterization of a new laccase from *Bacillus licheniformis* catalyzing dimerization of phenolic acids. *Appl. Microbiol. Biotechnol.* 79 217–224. 10.1007/s00253-008-1417-2 18330561

[B22] Kuppuraj S. P. Venkidasamy B. Selvaraj D. Ramalingam S. (2021). Comprehensive in silico and gene expression profiles of MnP family genes in *Phanerochaete chrysosporium* towards lignin biodegradation. *Int. Biodeterior. Biodegrad.* 157:105143. 10.1016/j.ibiod.2020.105143

[B23] Lee S. Kang M. Bae J. H. Sohn J. H. Sung B. H. (2019). Bacterial valorization of lignin: strains, enzymes, conversion pathways, biosensors, and perspectives. *Front. Bioeng. Biotechnol.* 7:209. 10.3389/fbioe.2019.00209 31552235 PMC6733911

[B24] Leonowicz A. Cho N. Luterek J. Wilkolazka A. Wojtas-Wasilewska M. Matuszewska A. (2001). Fungal laccase: properties and activity on lignin. *J. Basic Microbiol.* 41 185–227. 11512451 10.1002/1521-4028(200107)41:3/4<185::aid-jobm185>3.0.co;2-t

[B25] Li K. Xu F. Eriksson K. E. L. (1999). Comparison of fungal laccases and redox mediators in oxidation of a nonphenolic lignin model compound. *Appl. Environ. Microbiol.* 65 2654–2660. 10.1128/aem.65.6.2654-2660.1999 10347057 PMC91392

[B26] Li L. Xie T. Liu Z. Feng H. Wang G. (2018). Activity enhancement of CotA laccase by hydrophilic engineering, histidine tag optimization and static culture. *Protein Eng. Des. Sel.* 31 1–5. 10.1093/protein/gzx064 29301022

[B27] Liu Y. Luo G. Ngo H. H. Guo W. Zhang S. (2020). Advances in thermostable laccase and its current application in lignin-first biorefinery: a review. *Bioresour. Technol.* 298:122511. 10.1016/j.biortech.2019.122511 31839492

[B28] Lu X. Wang C. Li X. Zhao J. Zhao X. (2017). Studying nonproductive adsorption ability and binding approach of cellobiohydrolase to lignin during bioconversion of lignocellulose. *Energy Fuels* 31 14393–14400. 10.1021/acs.energyfuels.7b02427

[B29] Machczynski M. C. Vijgenboom E. Samyn B. Canters G. W. (2004). Characterization of SLAC: a small laccase from *Streptomyces coelicolor* with unprecedented activity. *Protein Sci.* 13 2388–2397. 10.1110/ps.04759104 15295117 PMC2280001

[B30] Majumdar S. Lukk T. Solbiati J. O. Bauer S. Nair S. K. Cronan J. E. (2014). Roles of small laccases from *Streptomyces* in lignin degradation. *Biochemistry* 53 4047–4058. 10.1021/bi500285t 24870309

[B31] Mann D. G. J. Labbé N. Sykes R. W. Gracom K. Kline L. Swamidoss I. M. (2009). Rapid assessment of lignin content and structure in switchgrass (*Panicum virgatum* L.) grown under different environmental conditions. *Bioenergy Res.* 2 246–256. 10.1007/s12155-009-9054-x

[B32] Mate D. M. Alcalde M. (2015). Laccase engineering: from rational design to directed evolution. *Biotechnol. Adv.* 33 25–40. 10.1016/j.biotechadv.2014.12.007 25545886

[B33] Mayer A. M. Staples R. C. (2002). Laccase: new functions for an old enzyme. *Phytochemistry* 60 551–565. 10.1016/s0031-9422(02)00171-1 12126701

[B34] Navaneetha S. Vivanco J. M. Decker S. R. Reardon K. F. (2011). Expression of industrially relevant laccases: prokaryotic style. *Trends Biotechnol.* 29 480–489. 10.1016/j.tibtech.2011.04.005 21640417

[B35] Perna V. Meyer A. S. Holck J. Eltis L. D. Eijsink V. G. Wittrup Agger J. (2019). Laccase-catalyzed oxidation of lignin induces production of H_2_O_2_. *ACS Sustainable Chem. Eng.* 8 831–841. 10.1021/acssuschemeng.9b04912

[B36] Prins A. Kleinsmidt L. Khan N. Kirby B. Kudanga T. Vollmer J. (2015). The effect of mutations near the T1 copper site on the biochemical characteristics of the small laccase from Streptomyces coelicolor A3(2). *Enzyme Microb. Technol.* 68 23–32. 10.1016/j.enzmictec.2014.10.003 25435502

[B37] Rahmanpour R. Bugg T. D. (2015). Characterisation of Dyp-type peroxidases from *Pseudomonas fluorescens* Pf-5: oxidation of Mn (II) and polymeric lignin by Dyp1B. *Arch. Biochem. Biophys.* 574 93–98. 10.1016/j.abb.2014.12.022 25558792

[B38] Riva S. (2006). Laccases: blue enzymes for green chemistry. *Trends Biotechnol.* 24 219–226. 10.1016/j.tibtech.2006.03.006 16574262

[B39] Santos R. B. Capanema E. A. Balakshin M. Y. Chang H. Jameel H. (2012). Lignin structural variation in hardwood species. *J. Agric. Food Chem.* 60 4923–4930. 10.1021/jf301276a 22533315

[B40] Sherif M. Waung D. Korbeci B. Mavisakalyan V. Flick R. Brown G. (2013). Biochemical studies of the multicopper oxidase (small laccase) from *Streptomyces coelicolor* using bioactive phytochemicals and site-directed mutagenesis. *Microb. Biotechnol.* 6 588–597. 10.1111/1751-7915.12068 23815400 PMC3918160

[B41] Singh G. Sharma P. Capalash N. (2009). Performance of an alkalophilic and halotolerant laccase from gamma-proteobacterium JB in the presence of industrial pollutants. *J. Gen. Appl. Microbiol.* 55 283–289. 10.2323/jgam.55.283 19700922

[B42] Sugawara K. Nishihashi Y. Narioka T. Yoshida T. Morita M. Sugano Y. (2017). Characterization of a novel DyP-type peroxidase from *Streptomyces avermitilis*. *J. Biosci. Bioeng.* 123 425–430. 10.1016/j.jbiosc.2016.12.001 28089379

[B43] Toscano M. D. De Maria L. Lobedanz S. Ostergaard L. H. (2013). Optimization of a small laccase by active-site redesign. *Chembiochem* 14 1209–1211. 10.1002/cbic.201300256 23775916

[B44] Trubitsina L. I. Tishchenko S. V. Gabdulkhakov A. G. Lisov A. V. Zakharova M. V. Leontievsky A. A. (2015). Structural and functional characterization of two-domain laccase from *Streptomyces viridochromogenes*. *Biochimie* 112 151–159. 10.1016/j.biochi.2015.03.005 25778839

[B45] Villaverde J. J. Li J. Ek M. Ligero P. Vega A. D. (2009). Native lignin structure of miscanthus x giganteus and its changes during acetic and formic acid fractionation. *J. Agric. Food Chem.* 57 6262–6270. 10.1021/jf900483t 19552425

[B46] Xie F. Zhang W. Gong S. Gu X. Lan X. Wu J. (2019). Investigating lignin from *Canna edulis ker* residues induced activation of alpha-amylase: kinetics, interaction, and molecular docking. *Food Chem*. 271 62–69. 10.1016/j.foodchem.2018.07.153 30236724

[B47] Xu L. Wei Z. Min Z. Peng X. Yu G. Teng M. (2007). Crystal structures of *E. coli* laccase CueO at different copper concentrations. *Biochem. Biophys. Res. Commun.* 354 21–26. 10.1016/j.bbrc.2006.12.116 17217912

[B48] Yang C. Lü X. (2021). *Composition of plant biomass and its impact on pretreatment in Advances in 2nd Generation of Bioethanol Production.* Amsterdam: Elsevier, 71–85.

[B49] Yang C. Yue F. Cui Y. Xu Y. Shan Y. Liu B. (2018). Biodegradation of lignin by *Pseudomo*nas sp. Q18 and the characterization of a novel bacterial DyP-type peroxidase. *J. Appl. Microbiol.* 45 913–927. 10.1007/s10295-018-2064-y 30051274

[B50] Yang C. X. Wang T. Gao L. N. Yin H. J. Lu X. (2017). Isolation, identification and characterization of lignin-degrading bacteria from Qinling. *China J. Appl. Microbiol.* 123 1447–1460. 10.1111/jam.13562 28801977

[B51] Yang X. Gu C. Lin Y. (2020). A novel fungal laccase from *Sordaria macrospora* k-hell: expression, characterization, and application for lignin degradation. *Bioprocess Biosyst. Eng.* 43 1133–1139. 10.1007/s00449-020-02309-5 32067135

[B52] Yang Y. Zhang X. Yin Q. Fang W. Fang Z. Wang X. (2015). A mechanism of glucose tolerance and stimulation of GH1 β-glucosidases. *Sci. Rep.* 5 1–12. 10.1038/srep17296 PMC4658561 26603650

[B53] Ye W. Liu J. Wang H. Wang J. Wang X. (2012). Cloning, expression, purification, and characterization of a glutamate-specific endopeptidase from Bacillus licheniformis. *Protein Expr. Purif*. 82 138–143. 10.1016/j.pep.2011.12.001 22202650

[B54] Youn H. D. Hah Y. C. Kang S. O. (1995). Role of laccase in lignin degradation by white-rot fungi. *FEMS Microbiol. Lett.* 132 183–188. 10.1111/j.1574-6968.1995.tb07831.x

[B55] Zhang Y. Li X. Hao Z. Xi R. Cai Y. Liao X. (2016). Hydrogen peroxide-resistant CotA and YjqC of *Bacillus altitudinis* spores are a promising biocatalyst for catalyzing reduction of sinapic acid and sinapine in rapeseed meal. *PloS One.* 11:158351. 10.1371/journal.pone.0158351 27362423 PMC4928806

[B56] Zheng Z. Li H. Li L. Shao W. (2012). Biobleaching of wheat straw pulp with recombinant laccase from the hyperthermophilic *Thermus thermophilus*. *Biotechnol. Lett.* 34 541–547. 10.1007/s10529-011-0796-0 22102060

[B57] Zhu D. Liang N. Zhang R. Ahmad F. Sun J. (2020). Insight into depolymerization mechanism of bacterial laccase for lignin. *ACS Sustain. Chem. Eng.* 8 12920–12933. 10.1021/acssuschemeng.0c03457

