# Supplementary material for: A Novel Polyphenol Oxidoreductase OhLac from Ochrobactrum sp. J10 for Lignin Degradation
Source: Front Microbiol. 2021 Oct 4;12:694166. doi: 10.3389/fmicb.2021.694166 (PMC8521193; doi:10.3389/fmicb.2021.694166)
Supplement: Supplementary file 1 [file Data_Sheet_1.pdf]

The sequence of OhLac:

SEQ ID No.1:

ATGAATACATATCACCCATTCAGTCTTACCACACCCTCGACACTCATGATACAAGACTG
GGCTCAAACGAATCAAAACAACAAAGAGGTCATTGCCGGATTTACGACAAAAAACGGCGG
TGTCAGCCAAAAGCCTTTTGAATCGTTAAATACAGGATTGCACGTTCATGACAAAGATGCA
GATGTAGTTAAAAATCGTGAATATATTGCCGATATGTTTAATACTGATTTGCAGTCTTGGGTAT
TCGCTGATCAGACACATGATAATCGCGTTCAGAAAGTGACGCAGAGGGATAGGGGAAAAG
GCGCCCGTGAGTATCACACGGCTCTAAAAGCAACGGACGGGCTCTATACAAATGAAAAAA
ATGTATTTTTAGCATTATGCTTTGCTGATTGTGTGCCTCTTTTCTTTTATGATCCGGTTCAGTC
GCTTGTCGGAGTCGCCCATGCCGGGTGGAAAGGCACCGTCAAACAGATTGGCAGAGAAAT
GGTGAAGCAATGGACTGAGAAGGAAGGTTCAAATCCCTCAGATATTTACGCTGTTATTGGC
CCGTCTATCAGCGGTGCATGCTATACGGTAGACGACCGCGTCATGGATGCTGTCCGCGCATT
GCCGGTTTCAGCAGACCTTGCCGTTAATCAGACGGCAAAGGCACAATATCAGCTTGATCTG
AAAGAGCTGAACCGTCTTATACTGATGGACAGCGGTTTGGCAAGTGAACAAATTTCTGTCA
GCGGTTTATGCACGGAAAGCGAGCCGTCTCTTTTCTATTCTCACCGCCGCGATCAGGGGAA
AACTGGACGGATGATGTCCTTTATCGGAATGAAGGAGGCATAA

SEQ ID No.2：

MNTYHPFSLTTPSTLMIQDWAQTNQNNKEVIAGFTTKNGGVSQKPFESLNTGLHVHDKD
ADVVKNREYIADMFNTDLQSWVFADQTHDNRVQKVTQRDRGKGAREYHTALKATDGLYTNE
KNVFLALCFADCVPLFFYDPVQSLVGVAHAGWKGTVKQIGREMVKQWTEKEGSNPSDIYAVI
GPSISGACYTVDDRVMDAVRALPVSADLAVNQTAKAQYQLDLKELNRLILMDSGLASEQISVS
GLCTESEPSLFYSHRRDQGKTGRMMSFIGMKEA-

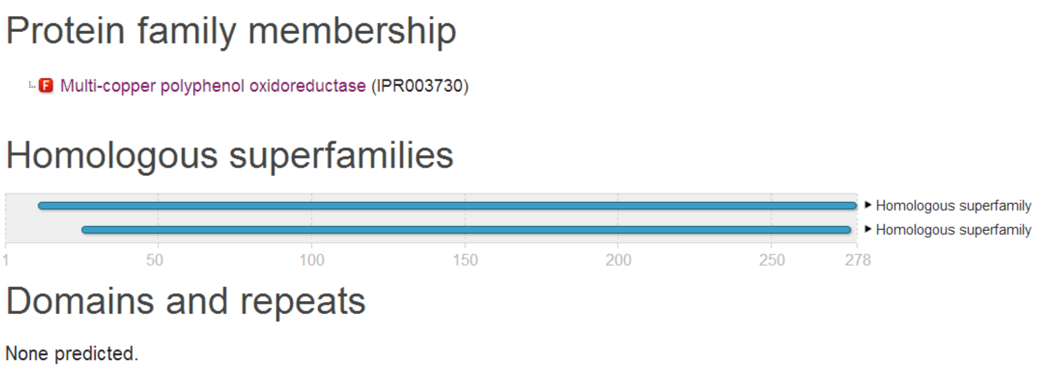

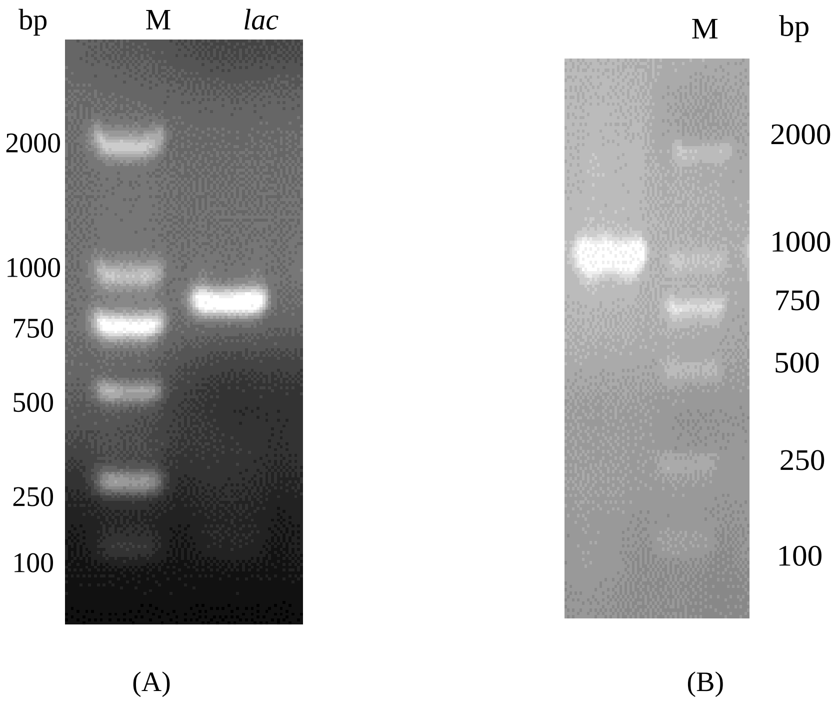

Results of PCR products. (A) The amplified fragment of small laccase *OhLac* gene；(B) The colony PCR verification results of recombinant engineering bacteria of OhLac
