# Supplementary figures and images for: A Novel Polyphenol Oxidoreductase OhLac from Ochrobactrum sp. J10 for Lignin Degradation
Source: Front Microbiol. 2021 Oct 4;12:694166. doi: 10.3389/fmicb.2021.694166 (PMC8521193; doi:10.3389/fmicb.2021.694166)

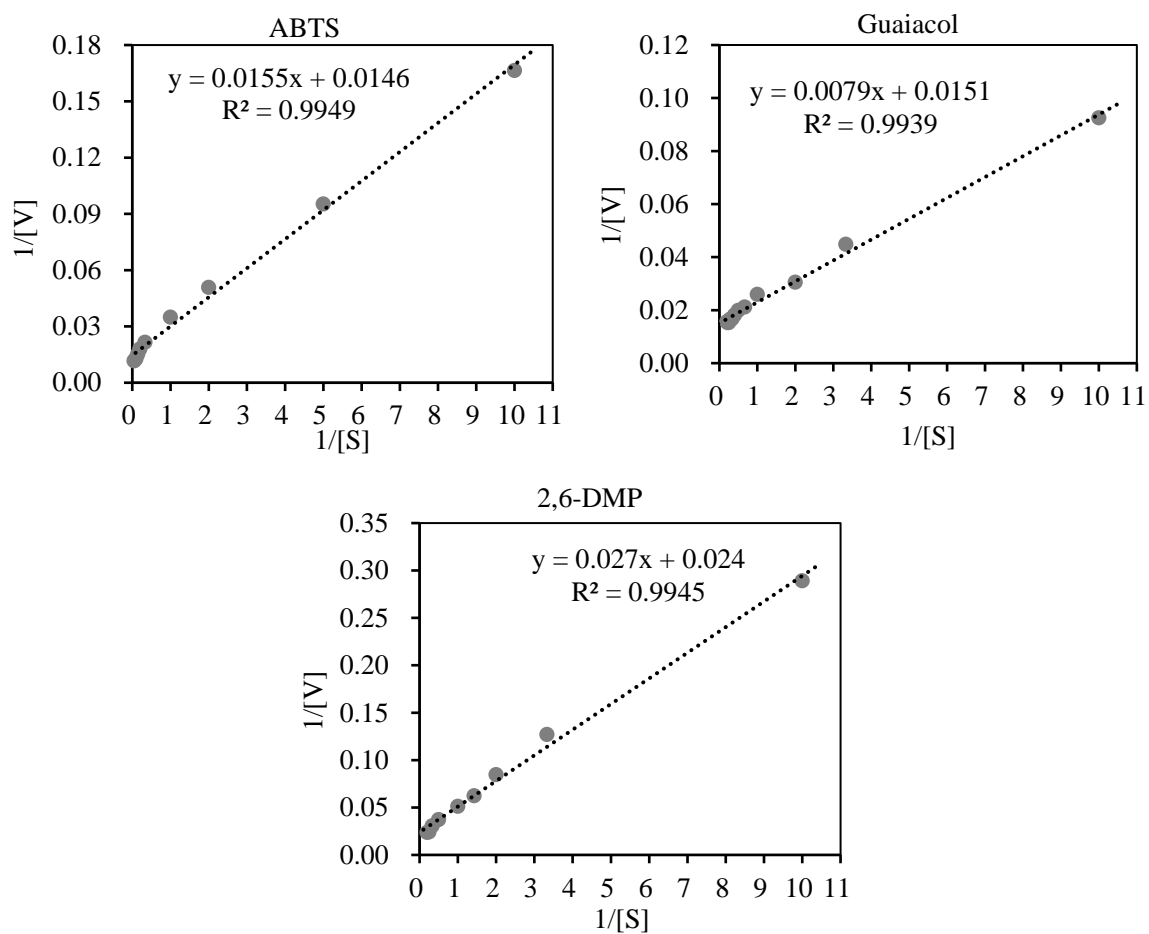

Fig. S2 The Lineweaver-Burk plot of the substrate concentration versus reaction rate

Supplement: Supplementary file 2 [file Data_Sheet_2.pdf]
