# Supplementary material for: A Novel Polyphenol Oxidoreductase OhLac from Ochrobactrum sp. J10 for Lignin Degradation
Source: Front Microbiol. 2021 Oct 4;12:694166. doi: 10.3389/fmicb.2021.694166 (PMC8521193; doi:10.3389/fmicb.2021.694166)
Supplement: Supplementary file 3 [file Data_Sheet_3.pdf]

Supplementary Figure Legends

Fig. S1. The bioinformation of OhLac from *Ochrobactrum* sp. J10.

Fig. S2. The steady-state kinetic parameters of OhLac.
