# Supplementary material for: A Novel Polyphenol Oxidoreductase OhLac from Ochrobactrum sp. J10 for Lignin Degradation
Source: Front Microbiol. 2021 Oct 4;12:694166. doi: 10.3389/fmicb.2021.694166 (PMC8521193; doi:10.3389/fmicb.2021.694166)
Supplement: Supplementary file 4 [file Table_1.docx]

**Table S1** Purification of OhLac from *Ochrobactrum* sp. J10.

| Purification step | Volume  (mL) | Activity  (U/mL） | Total activity （U） | Protein content  （mg/L) | Specific activity  (U/mg) | Purification  （fold) | Recovery Rate  (%) |
| --- | --- | --- | --- | --- | --- | --- | --- |
| Crude extract | 350 | 6.70 | 2345.00 | 281.90 | 23.77 | 1.00 | 100 |
| Purified OhLac by Ni-NTA | 61.30 | 13.49 | 827.12 | 76.20 | 177.03 | 7.45 | 35.27 |

The proteinof crude extract obtained from cultured cells by resuspending the *E. coli* in lysis buffer and lysing by ultrasonication.
